# Development of a machine learning-based prediction model for sepsis-associated delirium in the intensive care unit

**DOI:** 10.1038/s41598-023-38650-4

**Published:** 2023-08-04

**Authors:** Yang Zhang, Juanjuan Hu, Tianfeng Hua, Jin Zhang, Zhongheng Zhang, Min Yang

**Affiliations:** 1 The Second Department of Critical Care Medicine, The Second Affiliated Hospital of Anhui Medical University, Hefei, 230601 Anhui People’s Republic of China; 2 The Laboratory of Cardiopulmonary Resuscitation and Critical Care, The Second Affiliated Hospital of Anhui Medical University, Hefei, 230601 Anhui People’s Republic of China; 3 Department of Emergency Medicine, Sir Run Run Shaw Hospital, Zhejiang University School of Medicine, No. 3, Hangzhou, 310016 Zhejiang People’s Republic of China

**Keywords:** Risk factors, Data mining, Machine learning, Predictive medicine, Infectious diseases, Neurological disorders

## Abstract

Septic patients in the intensive care unit (ICU) often develop sepsis-associated delirium (SAD), which is strongly associated with poor prognosis. The aim of this study is to develop a machine learning-based model for the early prediction of SAD. Patient data were extracted from the Medical Information Mart for Intensive Care IV (MIMIC-IV) database and the eICU Collaborative Research Database (eICU-CRD). The MIMIC-IV data were divided into a training set and an internal validation set, while the eICU-CRD data served as an external validation set. Feature variables were selected using least absolute shrinkage and selection operator regression, and prediction models were built using logistic regression, support vector machines, decision trees, random forests, extreme gradient boosting (XGBoost), k-nearest neighbors and naive Bayes methods. The performance of the models was evaluated in the validation set. The model was also applied to a group of patients who were not assessed or could not be assessed for delirium. The MIMIC-IV and eICU-CRD databases included 14,620 and 1723 patients, respectively, with a median time to diagnosis of SAD of 24 and 30 h. Compared with Non-SAD patients, SAD patients had higher 28-days ICU mortality rates and longer ICU stays. Among the models compared, the XGBoost model had the best performance and was selected as the final model (internal validation area under the receiver operating characteristic curves (AUROC) = 0.793, external validation AUROC = 0.701). The XGBoost model outperformed other models in predicting SAD. The establishment of this predictive model allows for earlier prediction of SAD compared to traditional delirium assessments and is applicable to patients who are difficult to assess with traditional methods.

## Introduction

Sepsis is a severe organ dysfunction caused by a dysregulated host response to infection, with high incidence and mortality, and is a common critical illness^1^. Approximately 48 million people worldwide suffer from sepsis each year and approximately 11 million people die from it^2^. Delirium is the most common manifestation of brain dysfunction in critically ill patients, characterized by symptoms such as altered consciousness, impaired attention, disorientation, hallucinations and delusions^3^. Delirium is a common neurological complication in septic patients in the intensive care unit (ICU), with reported incidence rates ranging from 17.7 to 48%, and its severity is closely associated with patient prognosis^4,5^. Furthermore, sepsis-induced delirium is also associated with long-term cognitive dysfunction after discharge, causing physical discomfort and pain to patients and a burden to families and the economy^6,7^.

Sepsis-associated delirium (SAD) is a complex clinical syndrome, the mechanism of which is not fully understood. It may be related to several factors, including neuroinflammation, cerebral perfusion abnormalities, blood–brain barrier damage, and neurotransmitter imbalances^8^. Currently, there is no definitive diagnostic criterion for SAD, and the Confusion Assessment Method for the ICU (CAM-ICU) score is the most effective tool for diagnosing and assessing delirium in adult ICU patients according to the 2013 Society of Critical Care Medicine guidelines for pain, agitation, and delirium^9^. There is still no specific treatment for SAD, and early detection and prevention of SAD in septic patients are critical to its occurrence and prognosis^10^. Several studies have analyzed the risk factors for SAD in septic patients^4,5,11^, but there is still no early prediction tool for SAD in septic patients.

The aim of this study is to develop an early prediction model for SAD using machine learning methods based on sepsis-related data from large public databases and to evaluate the clinical applicability of this model. Our ultimate goal is to provide clinicians with a tool to identify high-risk patients more quickly and comprehensively, allowing for earlier implementation of preventive measures and ultimately reducing the incidence and mortality of SAD.

## Materials and methods

### Data source

This is a retrospective cohort study based on the Medical Information Mart for Intensive Care-IV (MIMIC-IV, version 2.2) and the eICU Collaborative Research Database (eICU-CRD, version 2.0)^12,13^. The MIMIC-IV database contains information on all patients admitted to Beth Israel Deaconess Medical Center between 2008 and 2019, while the eICU-CRD is a multicenter telemedicine database containing data from more than 200,000 patients admitted to 335 ICUs in 208 hospitals across the United States between 2014 and 2015. The database includes comprehensive information such as length of stay, laboratory tests, medication management, vital signs, etc. for each patient. To protect patient privacy, all personal information was de-identified and random codes were used instead of patient identifiers. Therefore, this study did not require patient consent or ethics approval. The researcher (Zhang) has completed the training program provided by the collaborating institution (Certificate No. 53496787) and is qualified to use the database and extract data.

### Study population

The diagnosis of sepsis was based on the Third International Consensus Definitions for Sepsis and Septic Shock (Sepsis-3), which defines sepsis as a sequential organ failure assessment (SOFA) score ≥ 2 associated with infection or suspected infection. Suspected infection was defined as antibiotics given within 3 days or 24 h of culture collection^1^. The following patients were excluded: (1) those aged < 18 years; (2) patients with multiple ICU admissions; (3) patients with an ICU stay of less than 24 h.

The presence of delirium was assessed using the CAM-ICU score, which consists of four features: (1) an acute onset of mental status changes or a fluctuating course; (2) inattention; (3) disorganized thinking; and (4) an altered level of consciousness. A patient is diagnosed as delirious (i.e., CAM-ICU positive) if they exhibit features 1 and 2, along with either feature 3 or 4^14^.

We excluded septic patients without documented delirium assessment and septic patients who could not be assessed (documented inability to assess any of the 4 characteristics of the CAM-ICU scale). In addition, patients with a positive delirium assessment before the onset of sepsis and outside the ICU were excluded.

### Data extraction and processing

The following data were extracted from the MIMIC-IV and eICU-CRD databases: (1) demographic information; (2) type of initial ICU admission; (3) initial vital signs and laboratory test results within 24 h of ICU admission; (4) SOFA and Glasgow Coma Scale (GCS) scores within 24 h of ICU admission; (5) comorbidities (hypertension, diabetes, acute myocardial infarction, chronic obstructive pulmonary disease, stroke, chronic kidney disease, acute kidney injury); (6) use of mechanical ventilation (MV), continuous renal replacement therapy (CRRT), vasopressors, and sedatives within 24 h of ICU admission; (7) ICU length of stay, 28-days ICU mortality, diagnosis time for delirium and sepsis. For continuous variables, outliers and obviously conflicting values were considered as missing values (for example, numerical values for vital signs were eliminated using certain rules (i.e., heart rate values should be between 0 and 300). Variables with more than 20% missing values were excluded from the analysis. Multiple imputation for missing values was performed using the “MICE” package^15^. For unordered multicategorical variables, one-hot coding was used to represent them.

### Statistical analysis

Continuous variables were expressed as median and interquartile range. The Mann–Whitney U test was used for statistical comparisons between two groups. Categorical variables were described as counts and percentages, and the Chi-squared test or Fisher's exact test was used for group comparisons. Kaplan–Meier survival curves were constructed and compared using the log-rank test.

MIMIC-IV data were randomly divided into training and internal validation sets in a 7:3 ratio, with eICU-CRD data serving as the external validation set. Least absolute shrinkage and selection operator (LASSO) regression was used for dimensionality reduction and feature selection^16^. After data reduction, predictive models were built using the following methods: (1) logistic regression (LR); (2) support vector machine (SVM); (3) decision tree (DT); (4) random forest (RF); (5) extreme gradient boosting (XGBoost); (6) k-nearest neighbors (KNN); and (7) naive bayes (NB).

Model performance was evaluated using area under the receiver operating characteristic curve (AUROC), specificity, sensitivity, positive predictive value (PPV), negative predictive value (NPV), accuracy, and kappa coefficient, with AUROC serving as the primary performance metric. We also evaluated the change in PPV and NPV of the model at different prevalence rates. The model with optimal predictive performance was selected as the primary model for this study. Calibration curves were used to assess the degree of agreement between observed and predicted outcomes, and decision curve analysis (DCA) was used to assess net clinical benefit.

The Shapley Additive Explanations (SHAP) method was used to explore the interpretability of the final predictive model. Higher SHAP values indicated an increased likelihood of SAD^17^. Partial dependence plots (PDPs) could be used to calculate SHAP values for each feature, allowing clinicians to make more accurate predictions. PDPs can show the marginal effects of each feature on the predictions of the machine learning model.

To evaluate the application of the model, we applied the final model to another group of patients in the MIMIC-IV database who were not assessed or could not be assessed for delirium and predicted the occurrence of SAD in these individuals.

All statistical analyses were performed using R 4.2.3 (Vienna, Austria) and STATA 15.1 (College Station, Texas), with *P* < 0.05 considered statistically significant. The machine learning code and the raw patient data are available on Github (https://github.com/bbycat927/SAD).

### Ethics approval and consent to participate

The MIMIC-IV database was approved by the Institutional Review Boards of Beth Israel Deaconess Medical Center and the Massachusetts Institute of Technology. Access to the eICU-CRD database was approved by the Institutional Review Board of the Massachusetts Institute of Technology. All protected health information in the database was de-identified, eliminating the need for individual patient consent. All methods were performed in accordance with relevant guidelines and regulations.

## Results

### Participants and baseline characteristics

**Figure 1:**
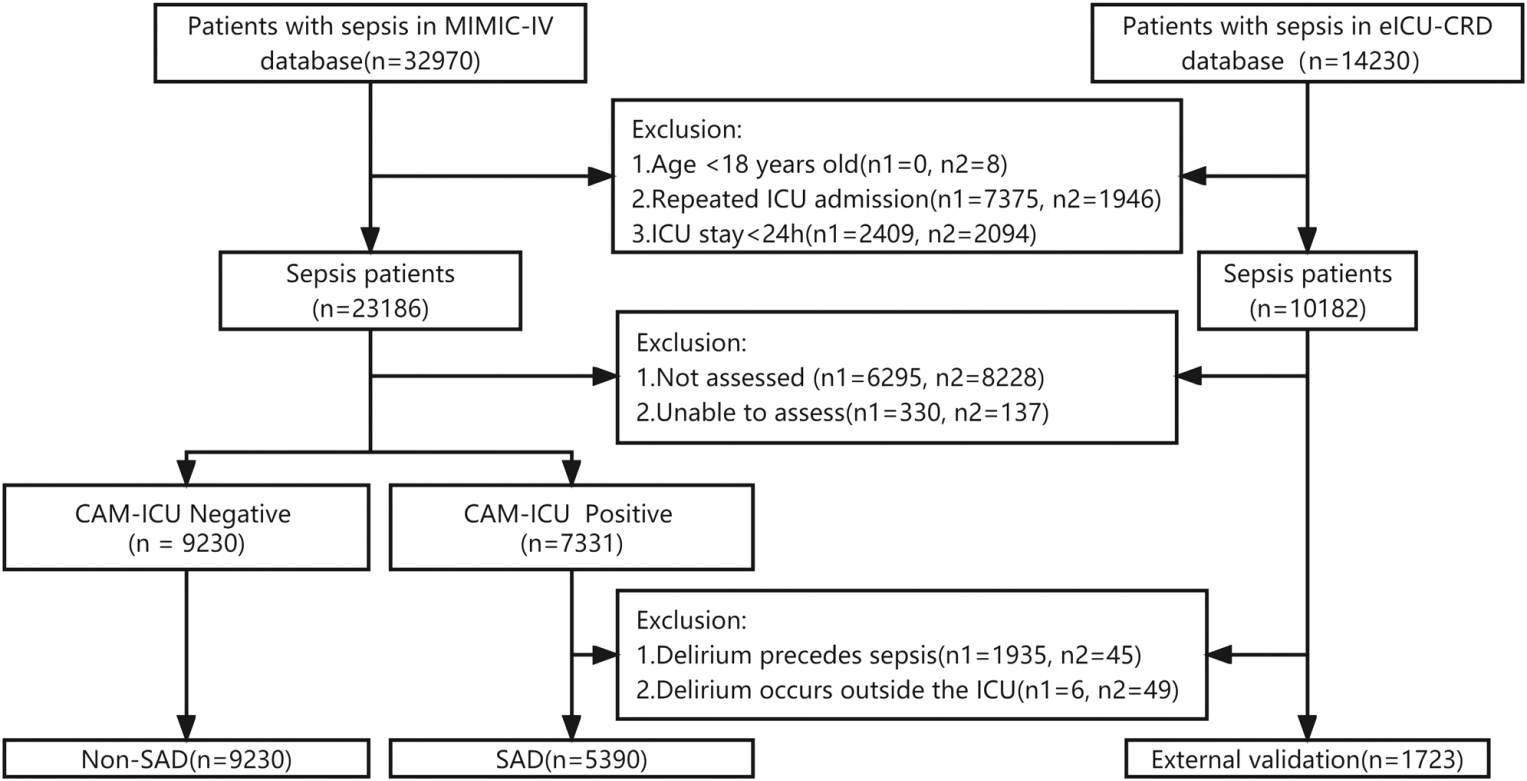
Research flowchart. n1, patients excluded in MIMIC-IV database. n2, patients excluded in eICU-CRD database.

**Figure 2:**
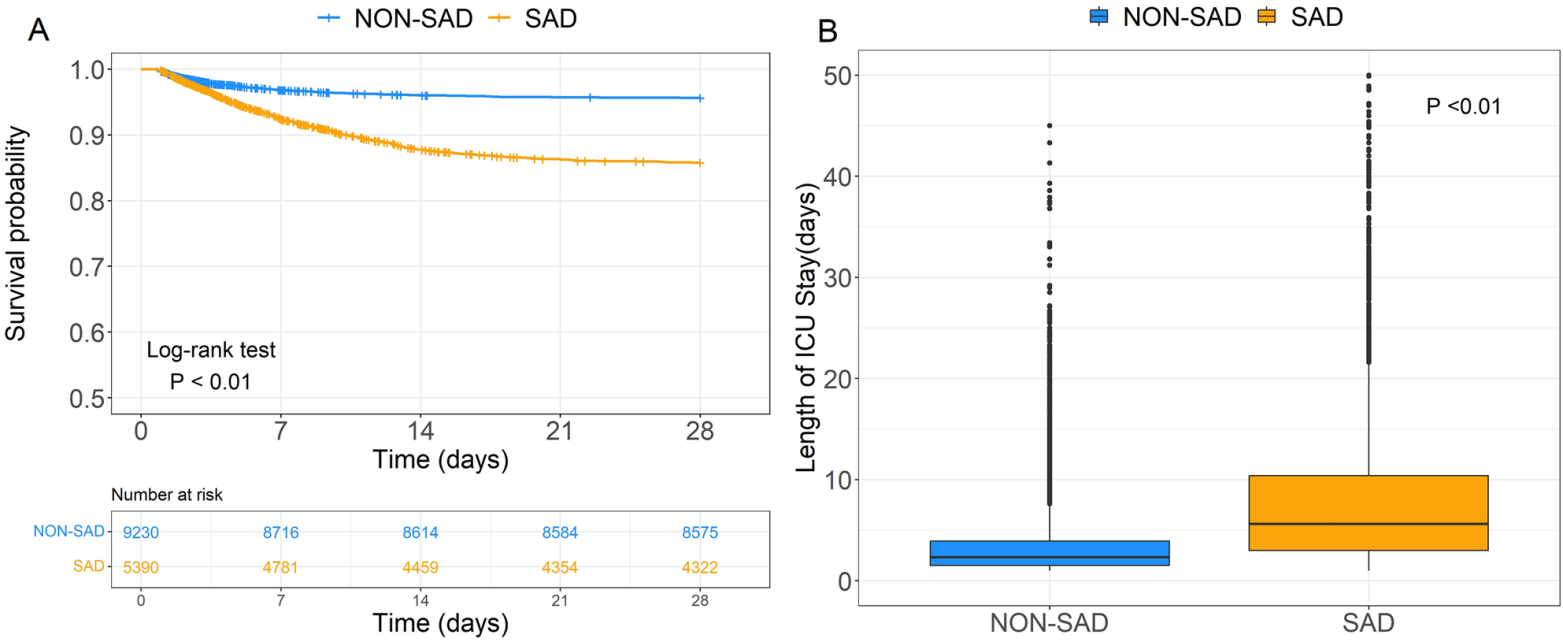
(**A**) Kaplan–Meier survival curves of 28-days ICU mortality for SAD and Non-SAD groups in the MIMIC-IV database. (**B**) Boxplots of ICU length of stay for SAD and Non-SAD groups in the MIMIC-IV database.

After applying the exclusion criteria, a total of 14,620 patients from the MIMIC-IV database and 1723 patients from the eICU-CRD database were included (Fig. 1). Baseline characteristics of all patients are shown in Table 1. In the MIMIC-IV database, there were 5,390 cases of SAD (36.9%). Figure 2A shows the Kaplan–Meier curves for the two groups, showing a higher 28-days ICU mortality rate for the SAD group compared to the Non-SAD group (*P* < 0.01, Log-rank test). Similarly, ICU length of stay was significantly longer in the SAD group compared to the Non-SAD group (Fig. 2B  *P* < 0.01, Mann–Whitney U test).

**Table 1:** Baseline Characteristics of SAD and Non-SAD Patients.

Variables	MIMIC-IV cohort		*p*	eICU cohort		*p*
	Non-SAD patients (n = 9230)	SAD patients (n = 5390)		Non-SAD patients (n = 1212)	SAD patient (n = 511)	
Age	68 (57, 79)	69 (57, 80)	0.03	66 (54, 78)	67 (54, 78)	0.57
Weight, (Kg)	80 (67.5, 95.2)	79.9 (66.2, 96.2)	0.55	79.8 (65.7, 98.7)	77.1 (63.8, 98.8)	0.18
Sex Male, n (%)	3814 (41.3)	2288 (42.4)	0.19	564 (46.5)	256 (50.1)	0.19
Ethnicity, n (%)			< 0.01			0.17
Asian	311 (3.4)	115 (2.1)		8 (0.7)	4 (0.8)	
Black	766 (8.3)	500 (9.3)		154 (12.7)	70 (13.7)	
Hispanic	360 (3.9)	197 (3.7)		138 (11.4)	43 (8.4)	
White	6372 (69)	3351 (62.2)		857 (70.7)	364 (71.2)	
Other	417 (4.5)	225 (4.2)		5 (0.4)	0 (0)	
Unknown	1004 (10.9)	1002 (18.6)		50 (4.1)	30 (5.9)	
ICU type, n (%)			< 0.01			0.25
CCU	881 (9.5)	485 (9)		175 (14.4)	90 (17.6)	
CVICU	2772 (30)	689 (12.8)		25 (2.1)	10 (2)	
MICU	1601 (17.3)	1477 (27.4)		89 (7.3)	40 (7.8)	
MICU/SICU	1780 (19.3)	926 (17.2)		810 (66.8)	312 (61.1)	
NICU	234 (2.5)	300 (5.6)		31 (2.6)	19 (3.7)	
SICU	1112 (12)	775 (14.4)		82 (6.8)	40 (7.8)	
TSICU	850 (9.2)	738 (13.7)		–	–	
Vital signs						
Temperature, (℃)	36.7 (36.4, 37)	36.8 (36.5, 37.2)	< 0.01	36.8 (36.5, 37.3)	36.9 (36.4, 37.2)	0.63
Heart rate, (min^−1^)	85 (75, 100)	90 (78, 106)	< 0.01	93 (79, 109)	94 (81, 112)	0.11
Respiratory rate, (min^−1^)	18 (15, 22)	20 (16, 24)	< 0.01	20 (17, 24)	21 (17, 26)	0.02
Spo2, (%)	99 (96, 100)	98 (95, 100)	< 0.01	98 (95, 100)	98 (95, 100)	0.75
Systolic BP, (mmHg)	117 (103, 134)	120 (103, 137)	< 0.01	112 (95, 129)	110 (96, 132.5)	0.45
Diastolic BP, (mmHg)	63 (54, 74)	66 (55, 78)	< 0.01	62 (51, 73)	61 (51, 74)	0.89
Mean arterial BP, (mmHg)	79 (69, 90)	80.5 (69, 93)	< 0.01	75 (64, 88)	75 (63, 90)	0.72
Laboratory tests						
WBC, (K/uL)	11.4 (7.9, 15.9)	12.2 (8.5, 17.1)	< 0.01	12.7 (8.1, 17.9)	12.9 (8.2, 19.3)	0.35
Hemoglobin, (g/dL)	10.1 (8.7, 11.7)	10.4 (8.7, 12.1)	< 0.01	10.3 (8.9, 12.1)	10.3 (8.9, 11.8)	0.95
Platelet, (K/uL)	170 (121, 238)	177 (120, 242)	0.05	193 (128, 265)	195 (130, 266)	0.76
BUN, (mg/dL)	19 (13, 31)	23 (15, 39)	< 0.01	25 (16, 41.2)	31 (18, 51)	< 0.01
Creatinine, (mg/dL)	1 (0.7, 1.5)	1.1 (0.8, 1.8)	< 0.01	1.2 (0.8, 2.1)	1.4 (0.9, 2.6)	< 0.01
Glucose, (mg/dL)	129 (106, 163)	138 (110, 179)	< 0.01	130 (103, 175.2)	133 (103, 180)	0.55
Sodium, (mEq/L)	137 (134, 140)	138 (135, 141)	< 0.01	138 (135, 141)	139 (136, 143)	< 0.01
Chloride, (mEq/L)	104 (100, 108)	104 (100, 108)	0.2	104 (99, 108)	105 (100, 109)	< 0.01
Potassium, (mEq/L)	4.2 (3.8, 4.7)	4.2 (3.7, 4.7)	0.03	4.1 (3.6, 4.5)	4 (3.6, 4.6)	0.71
Magnesium, (mg/dL)	2 (1.7, 2.3)	1.9 (1.7, 2.2)	< 0.01	1.8 (1.6, 2.1)	1.9 (1.6, 2.1)	0.23
Total calcium, (mg/dL)	8.2 (7.7, 8.7)	8.2 (7.7, 8.7)	0.3	8.1 (7.5, 8.7)	8.1 (7.4, 8.6)	0.13
Phosphate, (mg/dL)	3.5 (2.8, 4.2)	3.7 (2.9, 4.6)	< 0.01	3.4 (2.7, 4.4)	3.5 (2.7, 4.8)	0.04
INR	1.3 (1.2, 1.6)	1.3 (1.2, 1.7)	0.7	1.4 (1.2, 1.8)	1.4 (1.2, 1.9)	0.17
Prothrombin time, (s)	14.6 (12.9, 17.2)	14.5 (12.5, 18.1)	0.49	16.3 (14.8, 20.3)	16.9 (14.9, 20.7)	0.15
PTT, (s)	31 (27.4, 36.9)	31.4 (27.5, 39)	< 0.01	33.5 (29.6, 41)	35.6 (30.8, 44.2)	< 0.01
Bicarbonate, (mEq/L)	23 (20, 25)	22 (19, 25)	< 0.01	23 (19, 27)	22 (19, 25)	< 0.01
Anion gap, (mEq/L)	14 (11, 17)	15 (13, 18)	< 0.01	12 (8.8, 16)	13 (9, 17.2)	< 0.01
Score						
GCS	15 (15, 15)	15 (15, 15)	< 0.01	15 (13, 15)	13 (9, 15)	< 0.01
SOFA	3 (2, 4)	3 (2, 5)	< 0.01	6 (5, 9)	8 (6, 11)	< 0.01
Treatment measures						
MV, n (%)	3256 (35.3)	3341 (62)	< 0.01	442 (36.5)	296 (57.9)	< 0.01
CRRT, n (%)	78 (0.8)	178 (3.3)	< 0.01	7 (0.6)	5 (1)	0.35
Vasopressor, n (%)	4238 (45.9)	2900 (53.8)	< 0.01	303 (25)	183 (35.8)	< 0.01
Sedation, n (%)	4262 (46.2)	2396 (44.5)	0.05	163 (13.4)	111 (21.7)	< 0.01
Comorbidity						
AMI, n (%)	937 (10.2)	707 (13.1)	< 0.01	57 (4.7)	25 (4.9)	0.96
CKD, n (%)	1813 (19.6)	1127 (20.9)	0.07	120 (9.9)	47 (9.2)	0.72
COPD, n (%)	286 (3.1)	246 (4.6)	< 0.01	136 (11.2)	50 (9.8)	0.43
Hypertension, n (%)	4072 (44.1)	2226 (41.3)	< 0.01	141 (11.6)	58 (11.4)	0.93
Diabetes, n (%)	1753 (19)	905 (16.8)	< 0.01	242 (20)	96 (18.8)	0.62
AKI, n (%)	4689 (50.8)	3469 (64.4)	< 0.01	585 (48.3)	267 (52.3)	0.14
Stroke, n (%)	453 (4.9)	688 (12.8)	< 0.01	22 (1.8)	17 (3.3)	0.08

Continuous variables were expressed as median and interquartile range, the Mann–Whitney *U* test was used for statistical comparisons between two groups. Categorical variables were described as counts and percentages, and the Chi-squared test or Fisher's exact test was used for group comparisons.

*ICU* intensive care unit, *CCU* coronary care unit, *CVICU* cardiovascular ICU, *MICU* medical ICU, *SICU* surgical ICU, *NICU* neuro ICU, *TSICU* trauma-neuro surgical ICU, *BP* blood pressure, *WBC* white blood cell count, *BUN* blood urea nitrogen, *INR* international normalized ratio, *PTT* partial thromboplastin time, *GCS* glasgow coma scale, SOFA sequential organ failure assessment, *MV* mechanical ventilation, *CRRT* continuous renal replacement therapy, *AMI* acute myocardial infarction, *CKD* chronic kidney disease, *COPD* chronic obstructive pulmonary disease, *AKI* acute kidney injury.

Supplementary Table S1 shows that the median time to diagnosis of sepsis in the MIMIC-IV and eICU-CRD databases was 3 and 0 h, the median time to diagnosis of SAD was 24 and 30 h, and the mean time to diagnosis of SAD was 44.9 and 58.7 h.

### Feature selection and model development

**Figure 3:**
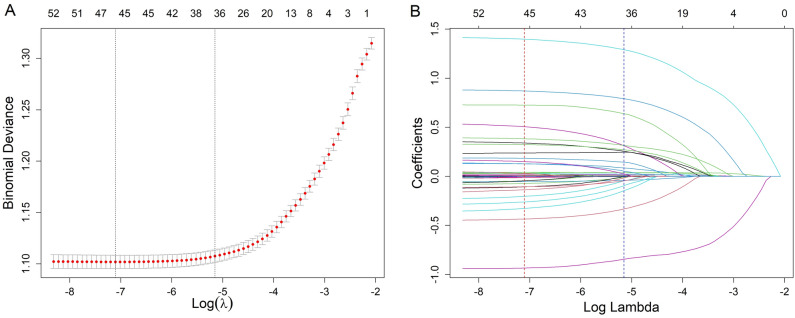
(**A**) Cross-validation plot for the penalty term. The dashed lines represent the lambda.min and lambda.1se. (**B**) Plots for the LASSO regression coefficients over different values of the penalty parameter. The vertical dashed lines correspond to the lambda.min and lambda.1se from the cross-validation.

Initially, 42 feature variables were identified (Table 1), and after one-hot coding of unordered multi-categorical variables, a total of 53 feature variables were obtained. LASSO regression was then performed. Figure 3A illustrates the cross-validation error for the penalty term. Using the lambda.1se criterion, we identified 43 variables with significant predictive ability. Figure 3B shows the coefficient profiles for these 53 features in LASSO regression, indicating the optimal point for retaining variables with non-zero coefficients. These 43 selected variables, along with their non-zero coefficient values, are presented in Supplementary Table S2. Based on the selected features, we built a traditional logistic regression model and six machine learning models: SVM, XGBoost, RF, KNN, DT, and NB.

### Model performance

**Figure 4:**
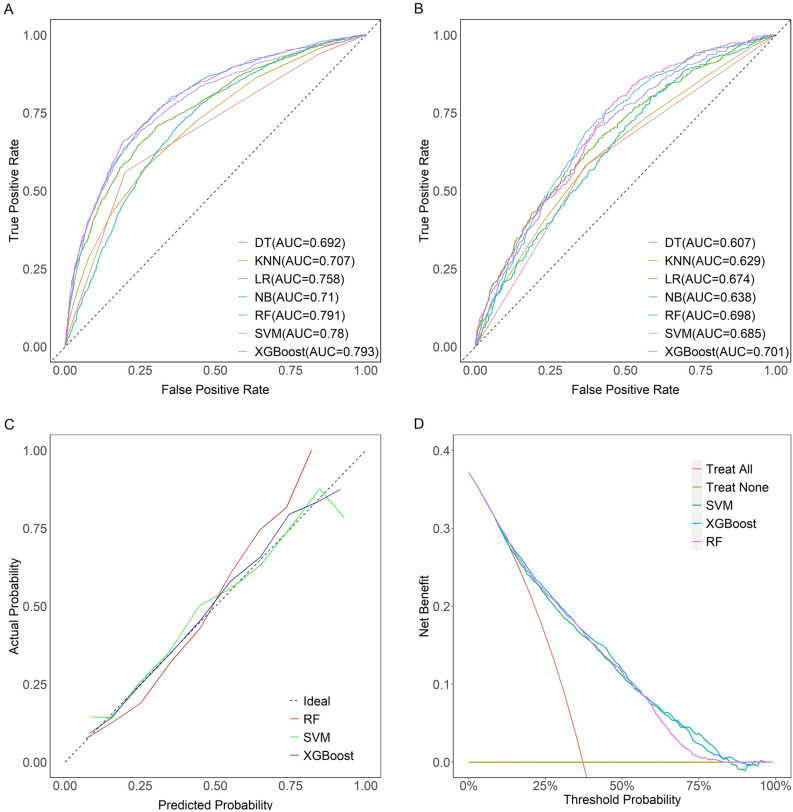
(**A**) The receiver operating characteristic (ROC) curves of the LR, SVM, XGBoost, RF, KNN, DT, and NB models on the internal validation set. (**B**) The ROC curves of the LR, SVM, XGBoost, RF, KNN, DT, and NB models on the external validation set. (**C**) Calibration curves of the XGBoost, RF, SVM models. (**D**) Decision curves of the XGBoost, RF, SVM models.

Table 2 describes the predictive performance of these models on the internal validation set, while Table 3 describes their performance on the external validation set. In terms of the AUROC, the XGBoost model outperformed the other models, with an AUROC of 0.793 on the internal validation set and 0.701 on the external validation set. The performance of the other models is also visualized in these figures, highlighting the superior performance of the XGBoost model (Fig. 4A and Fig. 4B).

**Table 2:** Model performance on the internal validation set.

Model	AUROC	Sensitivity	Specificity	PPV	NPV	Accuracy	Kappa
LR	0.758	0.8544	0.5058	0.7449	0.6729	0.7248	0.3795
SVM	0.780	0.8537	0.5463	0.7607	0.6886	0.7394	0.4176
RF	0.791	0.8530	0.5567	0.7647	0.6915	0.7428	0.4267
XGBoost	0.793	0.8515	0.5684	0.7692	0.6939	0.7462	0.4360
NB	0.710	0.8145	0.4476	0.7135	0.5882	0.6781	0.2755
DT	0.692	0.8004	0.5616	0.7551	0.6248	0.7116	0.3696
KNN	0.707	0.9220	0.2857	0.6856	0.6843	0.6854	0.2357

**Table 3:** Model performance on the external validation set.

Model	AUROC	Sensitivity	Specificity	PPV	NPV	Accuracy	Kappa
LR	0.674	0.7228	0.5205	0.7814	0.4419	0.6628	0.2314
SVM	0.685	0.6634	0.6086	0.8008	0.4325	0.6471	0.2433
RF	0.698	0.6856	0.5969	0.8014	0.4446	0.6593	0.2571
XGBoost	0.701	0.6642	0.5793	0.7892	0.4211	0.6390	0.2196
NB	0.638	0.6221	0.5616	0.7710	0.3852	0.6042	0.1623
DT	0.607	0.6353	0.5793	0.7817	0.4011	0.6187	0.1901
KNN	0.629	0.8812	0.2387	0.7330	0.4586	0.6907	0.1392

To examine the calibration of the models, calibration curves for the three best performing models (XGBoost, RF, SVM) were generated and compared (Fig. 4C). Among them, XGBoost showed the best fit between observed and predicted probabilities, indicating its superior calibration. Decision curve analysis (DCA) was performed on these three models and the results are shown in Fig. 4D. The analysis showed that using the XGBoost prediction model provided the highest net benefit for predicting SAD, outperforming both RF and SVM.

For further analysis, we evaluated the PPV and NPV of the models at different thresholds (prevalence rates). In the internal validation set, RF showed the highest PPV at a threshold of 0.3, while XGBoost and SVM maintained stable PPV with increasing thresholds. For the external validation set, RF and XGBoost showed superior PPV. However, XGBoost showed consistent PPV across all thresholds. While all models showed an increase in NPV with increasing thresholds, the NPV was generally lower compared to the internal validation set (Supplementary Tables S3 and S4). Overall, these results confirm the robustness of XGBoost, particularly its stability across different prevalence rates.

### Model interpretations

**Figure 5:**
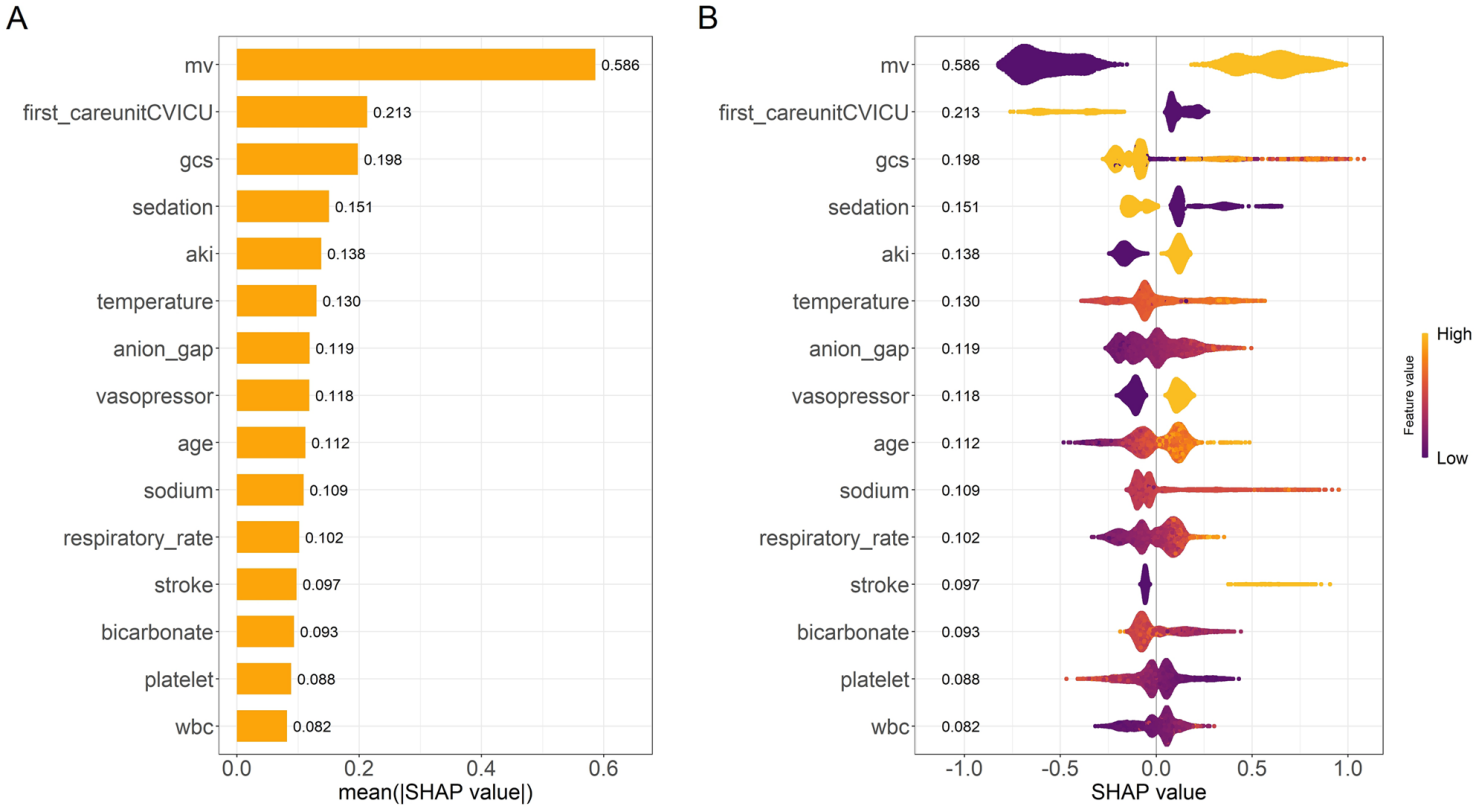
(**A**) Feature importance ranking plot of the XGBoost model (top 15 features). (**B**) SHAP summary plot of the XGBoost model (top 15 features). mv: mechanical ventilation, CVICU: cardiovascular ICU, wbc: white blood cell count, gcs: glasgow coma scale, aki: acute kidney injury.

To identify the most influential features in the model, we plotted the feature importance ranking for the XGBoost model (top 15 features, Fig. 5A). These features included mechanical ventilation, cardiovascular ICU (CVICU), GCS score, sedation, acute kidney injury (AKI), temperature, anion gap, blood sodium, vasopressors, respiratory rate, age, stroke, bicarbonate, platelets, and white blood cells. The SHAP Summary plot (Fig. 5B) complements this ranking by illustrating the impact of each feature on the model's output. Each dot on the plot corresponds to a SHAP value for a feature in a given case. The y-axis represents a feature, and the x-axis location indicates the SHAP value or the magnitude of the feature's effect on the prediction. The color of the dots represents the actual value of the feature, with purple indicating low values and yellow indicating high values (e.g., for MV, yellow dots on the right side of the zero line indicate higher MV values contributing to a higher risk of SAD).

**Figure 6:**
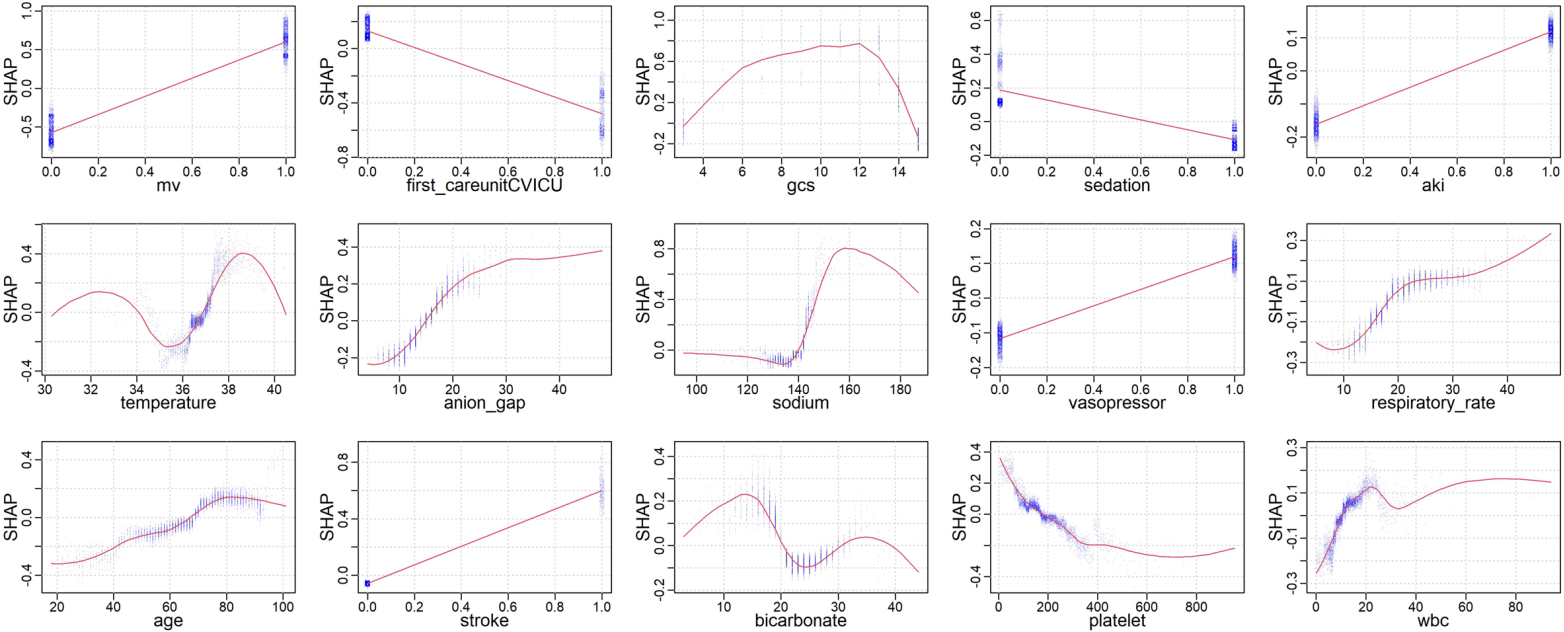
Partial dependence plots of features. Y-axis represents SHAP values; X-axis represents actual clinical parameters for continuous variables, and for binary variables (e.g., AKI, MV, sedation, stroke), ‘0’ indicates absence and ‘1’ indicates presence of the condition.

Partial Dependence Plots (PDPs) provide a graphical depiction of the marginal effect of a feature on the predicted outcome of a machine learning model (Fig. 6). In these plots, the x-axis represents the actual values of the clinical parameters, while the y-axis represents the corresponding SHAP values. This provides a way to quantify the relationship between the feature and the risk. A key advantage of PDPs is their ability to highlight non-linear relationships between features and the outcome. If the plotted line is not straight, or changes direction, this suggests that the relationship between the feature and the outcome is not linear. Thus, PDPs provide a more nuanced understanding of the model's decision rules beyond what is captured by linear models. For binary features, such as sedation, AKI, and stroke, the two distinct states of the variable are represented along the x-axis. The y-axis shows the average predicted outcome for the instances at each state. For example, a higher average prediction at one state over the other indicates that this state has a higher likelihood of leading to the predicted outcome. It's also worth noting that curve fitting for binary variables in PDPs does not indicate a trend or gradient as it does for continuous variables, but simply connects the average predictions at the two states.

### Application of the model

In the MIMIC-IV database, there were a total of 6625 patients who were either not assessed or unable to be assessed for delirium, with 330 patients falling into the latter category (Fig. 1). The baseline characteristics of these patients compared with those with sepsis included in the MIMIC-IV model are detailed in Supplementary Table S5. These patients had higher ICU 28-days mortality and in-hospital mortality compared with those in the model (*P* < 0.01). Using XGBoost model, we predicted the occurrence of SAD in these patients. In the total group, 1833 patients (27.7%) were predicted to develop SAD. Furthermore, when comparing patients who were unassessed and those who could not be assessed, we found a higher predicted SAD incidence rate in the latter group, at 44.5% compared to 26.8% in the former group (*P* < 0.01). Mortality rate and ICU length of stay were also higher in the group of patients who could not be assessed than in those who were unassessed (*P* < 0.01) (Supplementary Table S5).

## Discussion

In this investigation, we found that approximately 36.9% of sepsis patients in the ICU experienced delirium, with SAD patients having higher 28-days mortality rates and longer ICU stays compared to Non-SAD patients. We then developed an XGBoost-based machine learning predictive model that demonstrated commendable predictive performance in both internal and external validation, enabling early prediction of SAD on ICU admission. To our knowledge, this is the first study to establish a predictive model for SAD, as previous research has primarily focused on constructing predictive models for delirium^18–20^ or sepsis-associated encephalopathy^21–25^. Existing research on SAD has predominantly examined risk factors and typically included a limited number of study patients^4,5^.

Currently, the CAM-ICU score is the most commonly used method for diagnosing delirium, but it requires multiple assessments of the patient before a positive result is possible^9,14^. In contrast, our machine learning prediction model, based on data from the first 24 h of the patient's ICU admission, is able to predict SAD much earlier, as confirmed by our study results. It is worth noting that the completion of the delirium assessment by ICU staff (mainly nurses) varies widely, from only 38% in usual care to 84–95% after rigorous intervention^26^. Failure to complete has been attributed in part to patient-related factors such as age, language, sedation, and intubation, as well as staff-related issues such as inadequate training, difficulty using assessment tools, and heavy workload^27,28^. Even when an assessment is completed, a proportion of CAM-ICU scores are recorded as “unable to assess” (UTA) due to sedation, neurological deficits, underlying dementia or speech/hearing impairment. Such unassessable cases have been reported to account for 19–30% of all score records^26,29^. All of these factors can lead to underestimation of delirium in the ICU, and in our study we also found that many patients had no delirium assessment or were marked as UTA. Our predictive model revealed a SAD incidence of 27.7% in the cohort of unassessed patients, which was lower than the model's predicted incidence of 36.9%, while the SAD incidence in patients marked as UTA increased to a substantial 44.5%. Thus, by applying our machine learning prediction model to clinical data, clinicians may be able to identify potential SAD patients more comprehensively. However, it should be noted that further independent validation with different datasets with confirmed SAD diagnoses is needed to assess the generalizability and accuracy of this machine learning model in different clinical settings.

Our study identified mechanical ventilation as the strongest risk factor for SAD, with 50.6% of 6597 mechanically ventilated patients experiencing delirium, a finding consistent with many delirium-related studies^18,19^. In a study of mechanically ventilated sepsis patients, the incidence of SAD reached 48%^5^. In some partial dependence plots, we observed that sedation within 24 h of ICU admission was a favorable factor for SAD, which differs from some research findings^18^. Our sedatives included midazolam, dexmedetomidine, and propofol. Relevant studies have shown that the use of benzodiazepines and propofol may increase the risk of delirium^30,31^, whereas dexmedetomidine may decrease it^32^. However, the role of sedatives in SAD remains controversial; research by Yu Kawazoe et al.^33^ found no significant differences in mortality, delirium-free days, and ventilator-free days between the dexmedetomidine group and other sedative groups (propofol, midazolam, fentanyl) in mechanically ventilated sepsis patients. A large randomized controlled trial showed similar results^34^. We speculate that these results may be related to early sedation, as early sedation may reduce the duration of mechanical ventilation, which is the strongest risk factor for SAD, and its reduction would be conducive to reducing the incidence of SAD. Research by Stephens et al.^35^ found that the use of light sedation within the first 48 h of mechanical ventilation could reduce mortality, mechanical ventilation duration, and ICU length of stay. Shehabi et al.^36^ introduced the concept of early goal-directed sedation, implementing goal-directed sedation as soon as possible (12 h) after the initiation of mechanical ventilation, resulting in less benzodiazepine use, more delirium-free days, and less physical restraint in the early goal-directed therapy group. Notably, the impact of early sedation on patients is closely related to the depth of sedation; early deep sedation is associated with significantly increased rates of delirium, duration of mechanical ventilation, and mortality compared with early light sedation^35^.

Stroke is also a risk factor for SAD. Some of the current predictive models associated with delirium tend to exclude stroke from their exclusion criteria, possibly due to the difficulty in distinguishing overlapping symptoms between delirium and stroke. However, in recent years, there has been an increasing number of studies on delirium in stroke patients. A systematic review of delirium in neuro ICU(NICU) patients suggests the need for delirium assessment in stroke patients, with current tools being applicable for monitoring delirium in both stroke and brain injury patients^37^. The CAM-ICU score can accurately diagnose delirium after stroke, with a study by Mitasova et al. finding a sensitivity of 76%, specificity of 98%, and accuracy of 94% for the CAM-ICU in diagnosing delirium in stroke patients^38^. In addition, stroke-related delirium may interfere with the diagnosis of SAD, so we excluded pre-sepsis delirium in our exclusion criteria. Studies have shown that the incidence of delirium in stroke patients ranges from 10.7 to 16%^39,40^, while the incidence of delirium in the NICU ranges from 12 to 43%^37^. Infection is one of the risk factors for delirium in stroke patients^41^. The incidence of delirium is higher in sepsis patients with concomitant stroke; in our study, the incidence of delirium reached 50% in sepsis patients with stroke and 56.2% for SAD in the NICU.

Our results indicate that CVICU is a favorable factor for SAD, with an incidence rate of 19.9% in CVICU, similar to some studies^42^. The initial 24-h GCS score is also an important predictor of SAD, consistent with the results of the two most recent delirium prediction models^18,19^. Other predictive factors such as AKI, temperature, anion gap, blood sodium, vasopressors, respiratory rate, age, bicarbonate, platelets, and white blood cells have also been validated by similar studies or predictive models^19–25^. PDPs suggest that some of these predictors have a nonlinear relationship with the occurrence of SAD. For example, GCS score, temperature, sodium, and bicarbonate.

Our study has several limitations. First, there is currently no definitive diagnostic criterion for SAD. Although we established several inclusion and exclusion criteria, misdiagnosis and missed diagnoses remain inevitable. Second, we used LASSO regression for feature selection due to its efficiency in handling large numbers of variables, which may not be optimal for all models and may miss complex, non-linear relationships within the data. Third, it's important to note that the risk factor analysis based on PDPs may be subject to the assumption of feature independence. Finally, we did not further analyze the effects of sedative drug types, doses, and duration of use on SAD, which may complicate our predictive variables.

## Conclusion

SAD is common in ICU sepsis patients, with higher mortality rates and longer ICU stays than sepsis alone. Using our machine learning-based early prediction model, we can predict the risk of SAD earlier than delirium can be detected by traditional tools such as CAM-ICU, and this model can be applied to patients who are difficult to assess conventionally. The establishment of this model facilitates early risk identification and the implementation of preventive measures, potentially reducing the incidence and mortality of SAD.

### Supplementary Information

Supplementary Tables.

## Data Availability

Publicly available data sets were analyzed in this study. These data can be found here: https://physionet.org/about/database/.
