# Supplementary material for: Development of a machine learning-based prediction model for sepsis-associated delirium in the intensive care unit
Source: Sci Rep. 2023 Aug 4;13:12697. doi: 10.1038/s41598-023-38650-4 (PMC10403605; doi:10.1038/s41598-023-38650-4)
Supplement: Supplementary file 1 — Supplementary Tables. [file 41598_2023_38650_MOESM1_ESM.docx]

**Development of a machine learning-based prediction model for sepsis-associated delirium in the intensive care unit**

Yang Zhang ^1,2^, Juanjuan Hu ^1,2^, Tianfeng Hua^1, 2^, Jin Zhang ^1, 2^, Zhongheng Zhang^3^, Min Yang ^1, 2*^

* Correspondence: yangmin@ahmu.edu.cn

^1^The Second Department of Critical Care Medicine, The Second Affiliated Hospital of Anhui Medical University, Hefei, Anhui 230601, P. R. China

^2^The Laboratory of Cardiopulmonary Resuscitation and Critical Care, The Second Affiliated Hospital of Anhui Medical University, Hefei, Anhui 230601, P. R. China

^3^Department of Emergency Medicine, Sir Run Run Shaw Hospital, Zhejiang University School of Medicine, No. 3, Hangzhou, Zhejiang 310016, P. R. China.

Table S1: Diagnosis time for delirium and sepsis in the MIMIC-IV and eICU-CRD cohorts

| **Events** | **MIMIC-IV cohort**  **(n = 14620)** | **eICU-CRD cohort**  **(n = 1723)** | **P** |
| --- | --- | --- | --- |
| Delirium diagnosis time, (hour) | 24 (10, 55) | 30 (8.1, 75.3) | 0.1 |
| Sepsis diagnosis time, (hour) | 3 (2, 6) | 0 (0, 1) | < 0.01 |

Continuous variables were expressed as median and interquartile range, the Mann-Whitney U test was used for statistical comparisons between two groups*.*

Table S2: Variables selected by Lasso regression and coefficients of variables (β)

| **Variables** | **Coefficient (β)** | **Variables** | **Coefficient (β)** |
| --- | --- | --- | --- |
| Intercept | -8.5498846131 |  |  |
| Age | 0.0055640742 | CRRT | 0.3119329407 |
| Weight | -0.0001182091 | Vasopressor | 0.2550309135 |
| Temperature | 0.0853413193 | Sedation | -0.3329321779 |
| Heart Rate | 0.0024972940 | SOFA | 0.0365017692 |
| Resp Rate | 0.0188996564 | AMI | 0.0544255914 |
| Spo2 | -0.0030585699 | CKD | -0.0459868675 |
| Diastolic BP | 0.0014288596 | COPD | 0.0236483893 |
| WBC | 0.0017266608 | Hypertension | -0.0006874155 |
| Platelet | -0.0005014600 | Diabetes | -0.0463030261 |
| BUN | 0.0031183116 | AKI | 0.3148988654 |
| Creatinine | -0.0118585143 | Stroke | 0.7907045565 |
| Glucose | 0.0005944812 | Race: AISAN | -0.1472491623 |
| Sodium | 0.0228005939 | Race: unknown | 0.2708038791 |
| Prothrombin time | 0.0002721136 | ICU type: CCU | -0.0952969295 |
| PTT | 0.0006458132 | ICU type: CVICU | -0.8459212571 |
| Bicarbonate | -0.0013227827 | ICU type: MICU | 0.2450186848 |
| Anion gap | 0.0370296318 | ICU type: MICU/SICU | -0.0162668751 |
| GCS | -0.0412995636 | ICU type: NICU | 0.6382943931 |
| MV | 1.2901092934 | ICU type: TSICU | 0.1442200572 |

These coefficients are calculated at a given lambda value, which is selected by cross-validation to minimize the sum of squared errors of the model. The larger the absolute value of the coefficient of a variable, the greater the effect of that variable on the predicted outcome. A coefficient of 0 for a variable is rejected by the Lasso regression, i.e., it is considered to have little effect on the prediction results.

Table S3: PPV and NPV values for model at different thresholds in the internal validation set

|  | PPV | | | | |  | NPV | | | | |
| --- | --- | --- | --- | --- | --- | --- | --- | --- | --- | --- | --- |
|  | 0.3 | 0.4 | 0.5 | 0.6 | 0.7 |  | 0.3 | 0.4 | 0.5 | 0.6 | 0.7 |
| LR | 0.81 | 0.78 | 0.74 | 0.71 | 0.68 |  | 0.53 | 0.61 | 0.67 | 0.73 | 0.84 |
| SVM | 0.81 | 0.79 | 0.76 | 0.73 | 0.69 |  | 0.59 | 0.65 | 0.69 | 0.74 | 0.80 |
| RF | 0.86 | 0.81 | 0.76 | 0.70 | 0.65 |  | 0.54 | 0.61 | 0.69 | 0.77 | 0.83 |
| XGBoost | 0.83 | 0.81 | 0.77 | 0.73 | 0.69 |  | 0.58 | 0.64 | 0.69 | 0.75 | 0.81 |
| NB | 0.73 | 0.72 | 0.71 | 0.70 | 0.70 |  | 0.57 | 0.58 | 0.59 | 0.59 | 0.59 |
| KNN | 0.75 | 0.71 | 0.69 | 0.66 | 0.65 |  | 0.55 | 0.62 | 0.68 | 0.73 | 0.80 |

Table S4: PPV and NPV values for model at different thresholds in the external validation set

|  | PPV | | | | |  | NPV | | | | |
| --- | --- | --- | --- | --- | --- | --- | --- | --- | --- | --- | --- |
|  | 0.3 | 0.4 | 0.5 | 0.6 | 0.7 |  | 0.3 | 0.4 | 0.5 | 0.6 | 0.7 |
| LR | 0.83 | 0.80 | 0.78 | 0.76 | 0.74 |  | 0.38 | 0.41 | 0.44 | 0.49 | 0.53 |
| SVM | 0.85 | 0.83 | 0.80 | 0.78 | 0.74 |  | 0.38 | 0.41 | 0.43 | 0.46 | 0.47 |
| RF | 0.89 | 0.85 | 0.80 | 0.74 | 0.71 |  | 0.36 | 0.40 | 0.44 | 0.48 | 0.58 |
| XGBoost | 0.88 | 0.85 | 0.79 | 0.76 | 0.73 |  | 0.40 | 0.42 | 0.42 | 0.50 | 0.59 |
| NB | 0.78 | 0.77 | 0.77 | 0.77 | 0.76 |  | 0.38 | 0.38 | 0.39 | 0.39 | 0.39 |
| KNN | 0.78 | 0.75 | 0.73 | 0.72 | 0.71 |  | 0.40 | 0.43 | 0.46 | 0.53 | 0.48 |

The prediction probabilities of the decision tree (DT) model are mainly distributed around specific values (e.g., 0.2-0.3 and 0.6-0.7 in this study), so adjusting the thresholds has a limited impact on the PPV and NPV.

Table S5: Baseline comparisons of different patient groups

| **Variables** | **Not assessed and**  **unable to assess**  **(n = 6625)** | **Modeled**  **patients**  **(n = 14620)** | **P** | **Unable to assess**  **(n=330)** | **Not assessed**  **(n=6295)** | **P** |
| --- | --- | --- | --- | --- | --- | --- |
| Age | 67 (55.3, 78.6) | 68 (57, 79) | < 0.01 | 68.9 (56.6, 81.1) | 66.9 (55.2, 78.4) | 0.12 |
| Weight, (Kg) | 80 (67.1, 95.1) | 80 (67, 95.5) | 0.71 | 77.3 (63.8, 94) | 80 (67.4, 95.1) | 0.02 |
| Sex Male, n (%) | 3875 (58.5) | 6102 (41.7) | < 0.01 | 177 (53.6) | 3698 (58.7) | 0.08 |
| **Ethnicity, n (%)** |  |  | < 0.01 |  |  | < 0.01 |
| Asian | 185 (2.8) | 426 (2.9) |  | 23 (7) | 162 (2.6) |  |
| Black | 525 (7.9) | 1266 (8.7) |  | 31 (9.4) | 494 (7.8) |  |
| Hispanic | 232 (3.5) | 557 (3.8) |  | 24 (7.3) | 208 (3.3) |  |
| White | 4765 (71.9) | 9723 (66.5) |  | 174 (52.7) | 4591 (72.9) |  |
| Other | 153 (2.3) | 642 (4.4) |  | 13 (3.9) | 140 (2.2) |  |
| Unknown | 765 (11.5) | 2006 (13.7) |  | 65 (19.7) | 700 (11.1) |  |
| **ICU type, n (%)** |  |  | < 0.01 |  |  | < 0.01 |
| CCU | 584 (8.8) | 1366 (9.3) |  | 24(7.3) | 560(8.9) |  |
| CVICU | 1491 (22.5) | 3461 (23.7) |  | 61(18.5) | 1430(22.7) |  |
| MICU | 1462 (22.1) | 3078 (21.1) |  | 66(20) | 1396(22.2) |  |
| MICU/SICU | 1268 (19.1) | 2706 (18.5) |  | 57(17.3) | 1211(19.2) |  |
| NICU | 27 (0.4) | 534 (3.7) |  | 0(0) | 27(0.4) |  |
| SICU | 1026 (15.5) | 1887 (12.9) |  | 63(19.1) | 963(15.3) |  |
| TSICU | 767 (11.6) | 1588 (10.9) |  | 59(17.9) | 708(11.2) |  |
| **Vital signs** |  |  |  |  |  |  |
| Temperature, (℃) | 36.5 (35.9, 37.1) | 36.7 (36.4, 37.1) | < 0.01 | 36.6 (36.3, 37.1) | 36.5 (35.9, 37.1) | < 0.01 |
| Heart rate, (min-1) | 88 (76, 102) | 87 (76, 102) | 0.22 | 87 (75.2, 104) | 88 (76, 102) | 0.88 |
| Respiratory rate, (min-1) | 18 (14, 22) | 18 (15, 23) | < 0.01 | 18 (15, 22) | 18 (14, 22) | 0.04 |
| Spo2, (%) | 99 (96, 100) | 98 (95, 100) | < 0.01 | 99 (96, 100) | 99 (96, 100) | 0.5 |
| Diastolic BP, (mmHg) | 64 (54, 75) | 64 (55, 76) | 0.01 | 64 (55, 74) | 64 (54, 75) | 0.85 |
| **Laboratory tests** |  |  |  |  |  |  |
| WBC, (K/uL) | 11.6 (8.1, 16) | 11.7 (8.1, 16.3) | 0.24 | 11.9 (7.8, 16.5) | 11.6 (8.1, 15.9) | 0.59 |
| Platelet, (K/uL) | 191 (137, 261) | 172 (121, 239) | < 0.01 | 189.5 (132, 247.2) | 191 (137, 261) | 0.3 |
| BUN, (mg/dl) | 20 (14, 33) | 20 (14, 34) | 0.07 | 21 (14, 33) | 20 (14, 33) | 0.51 |
| Creatinine, (mg/dl) | 1 (0.7, 1.5) | 1 (0.7, 1.6) | 0.39 | 1 (0.8, 1.5) | 1 (0.7, 1.5) | 0.83 |
| Glucose, (mg/dl) | 133 (108, 171) | 131.5 (107, 169) | 0.13 | 142 (113.5, 179.2) | 133 (108, 170) | 0.01 |
| Sodium, (mEq/L) | 138 (135, 141) | 137 (134, 140) | < 0.01 | 138 (135.2, 142) | 138 (135, 141) | 0.08 |
| Prothrombin time, (s) | 15 (13.6, 17.1) | 14.6 (12.8, 17.4) | < 0.01 | 14.4 (12.9, 17) | 15 (13.6, 17.1) | < 0.01 |
| PTT, (s) | 31.5 (27.1, 38.8) | 31.1 (27.4, 37.6) | 0.35 | 31.6 (27.3, 38.2) | 31.5 (27.1, 38.8) | 0.82 |
| Bicarbonate, (mEq/L) | 23 (20, 26) | 22 (20, 25) | < 0.01 | 23 (19, 25) | 23 (20, 26) | < 0.01 |
| Anion gap, (mEq/L) | 13 (11, 16) | 14 (12, 17) | < 0.01 | 14.5 (12, 18) | 13 (11, 16) | < 0.01 |
| Score |  |  |  |  |  |  |
| GCS | 15 (15, 15) | 15 (15, 15) | 0.02 | 15 (14, 15) | 15 (15, 15) | < 0.01 |
| SOFA | 3 (2, 4) | 3 (2, 4) | 0.17 | 3 (2, 5) | 3 (2, 4) | 0.02 |
| Treatment measures |  |  |  |  |  |  |
| MV, n (%) | 3497 (52.8) | 6597 (45.1) | < 0.01 | 216 (65.5) | 3281 (52.1) | < 0.01 |
| CRRT, n (%) | 133 (2) | 256 (1.8) | 0.22 | 8 (2.4) | 125 (2) | 0.72 |
| Vasopressor, n (%) | 2990 (45.1) | 7138 (48.8) | < 0.01 | 179 (54.2) | 2811 (44.7) | < 0.01 |
| Sedation, n (%) | 3613 (54.5) | 6658 (45.5) | < 0.01 | 197 (59.7) | 3416 (54.3) | 0.06 |
| **Comorbidity** |  |  |  |  |  |  |
| AMI, n (%) | 625 (9.4) | 1644 (11.2) | < 0.01 | 24 (7.3) | 601 (9.5) | 0.2 |
| CKD, n (%) | 1020 (15.4) | 2940 (20.1) | < 0.01 | 43 (13) | 977 (15.5) | 0.25 |
| COPD, n (%) | 176 (2.7) | 532 (3.6) | < 0.01 | 15 (4.5) | 161 (2.6) | 0.04 |
| Hypertension, n (%) | 2898 (43.7) | 6298 (43.1) | 0.37 | 166 (50.3) | 2732 (43.4) | 0.02 |
| Diabetes, n (%) | 1577 (23.8) | 2658 (18.2) | < 0.01 | 101 (30.6) | 1476 (23.4) | < 0.01 |
| AKI, n (%) | 3832 (57.8) | 8158 (55.8) | < 0.01 | 208 (63) | 3624 (57.6) | 0.06 |
| Stroke, n (%) | 578 (8.7) | 1141 (7.8) | 0.02 | 49 (14.8) | 529 (8.4) | < 0.01 |
| **Events** |  |  |  |  |  |  |
| SAD, n (%) | 1833 (27.7) | 5390 (36.9) | < 0.01 | 147 (44.5) | 1686 (26.8) | < 0.01 |
| In-hospital mortality, n (%) | 1294 (19.5) | 1852 (12.7) | < 0.01 | 111 (33.6) | 1183 (18.8) | < 0.01 |
| Length of ICU stay, (hour) | 71 (41, 142) | 73 (43, 145) | < 0.01 | 83 (47.2, 157) | 70 (41, 141) | < 0.01 |
| ICU 28-day mortality, n (%) | 988 (14.9) | 1133 (7.7) | < 0.01 | 87 (26.4) | 901 (14.3) | < 0.01 |

Continuous variables were expressed as median and interquartile range, the Mann-Whitney U test was used for statistical comparisons between two groups. Categorical variables were described as counts and percentages, and the Chi-squared test or Fisher's exact test was used for group comparisons.
